# Response of nitric and nitrous oxide fluxes to N fertilizer application in greenhouse vegetable cropping systems in southeast China

**DOI:** 10.1038/srep20700

**Published:** 2016-02-05

**Authors:** Yaojun Zhang, Feng Lin, Yaguo Jin, Xiaofei Wang, Shuwei Liu, Jianwen Zou

**Affiliations:** 1 Jiangsu Key Laboratory of Low Carbon Agriculture and GHGs Mitigation, Nanjing Agricultural University, Nanjing 210095, China; 2 Department of Environmental Sciences, Centre for Carbon, Water and Food, The University of Sydney, New South Wales 2570, Australia

## Abstract

It is of great concern worldwide that active nitrogenous gases in the global nitrogen cycle contribute to regional and global-scale environmental issues. Nitrous oxide (N_2_O) and nitric oxide (NO) are generally interrelated in soil nitrogen biogeochemical cycles, while few studies have simultaneously examined these two gases emission from typical croplands. Field experiments were conducted to measure N_2_O and NO fluxes in response to chemical N fertilizer application in annual greenhouse vegetable cropping systems in southeast China. Annual N_2_O and NO fluxes averaged 52.05 and 14.87 μg N m^−2^ h^−1^ for the controls without N fertilizer inputs, respectively. Both N_2_O and NO emissions linearly increased with N fertilizer application. The emission factors of N fertilizer for N_2_O and NO were estimated to be 1.43% and 1.15%, with an annual background emission of 5.07 kg N_2_O-N ha^−1^ and 1.58 kg NO-N ha^−1^, respectively. The NO-N/N_2_O-N ratio was significantly affected by cropping type and fertilizer application, and NO would exceed N_2_O emissions when soil moisture is below 54% WFPS. Overall, local conventional input rate of chemical N fertilizer could be partially reduced to attain high yield of vegetable and low N_2_O and NO emissions in greenhouse vegetable cropping systems in China.

It is of great concern worldwide that active nitrogenous gases in the global nitrogen cycle contribute to regional and global-scale environmental issues^1^. Nitrous oxide (N_2_O) is an important long-lived greenhouse gas that contributes to global warming. Both N_2_O and nitric oxide (NO) play major roles in atmospheric chemistry processes, in which they are involved in the destruction of stratospheric ozone^2^,^3^,^4^. Agricultural activities are responsible for about 60% and 10% of global anthropogenic N_2_O and NO sources, respectively, largely due to fertilizer application increased in croplands^5^. The estimates of N_2_O and NO emissions from croplands have large uncertainties since the sources and sinks of N_2_O and NO are not well characterized in different agroecosystems (e.g. rice paddies, grain upland croplands, vegetable cropping systems)^6^,^7^,^8^. In addition, N_2_O and NO are generally interrelated in soil nitrogen biogeochemical cycles, and thus it is important to simultaneously examine these two gases emission from typical croplands^9^.

In recent years, vegetable production has become economically important in China, with its harvest area accounting for 45% of the world total^10^. Meanwhile, greenhouse vegetable cultivation has increased rapidly since 2000 and has reached to more than 3.44 million hectares in 2010^11^,^12^, accounting for 18.1% and 2.1% of the total vegetable and agricultural area, respectively^13^. It is common that excessive N fertilizer and frequent irrigation are adopted to maintain high yield in greenhouse vegetable fields by the local farmers in China. For example, annual N fertilization application rates are mostly around 1000–1500 kg N ha^−1^ in the greenhouse vegetable systems^14^,^15^, and even more than 2800 kg N ha^−1^ in some areas of China^16^,^17^. Indeed, excessive N fertilizer application with low N use efficiency in intensively vegetable fields in China has been of great concern with respect to agricultural and environmental issues^18^,^19^.

Vegetable cropping system has been recognized to be an important source of N_2_O and NO emissions^9^,^11^,^20^,^21^. Although greenhouse vegetable production typically constitutes multiple vegetable cropping rotations within a year, most field N_2_O and NO measurements were taken only within a certain individual vegetable cropping season, which limited insights into annual N_2_O and NO budgets due to their high inter-seasonal variations^10^,^22^,^23^. Therefore, field measurements of N_2_O and NO fluxes taken over a whole annual cycle would be particularly important to gain an insight into annual direct N_2_O and NO emissions from Chinese vegetable cropping systems^19^,^23^.

High N application can stimulate nitrification and/or denitrification processes and thus promote N_2_O and NO emissions from croplands^9^,^20^,^24^. In general, there is a strong increase of both N_2_O and NO emissions accompanying with N application rates in croplands^2^,^25^. Hoben *et al.*^26^ found a nonlinear exponentially increasing N_2_O response to N application rates from a corn-soybean rotation, while N_2_O emissions were not significantly reduced with decreasing nitrogen fertilizer application in a winter wheat-summer maize rotation cropland by Yan *et al.*^27^. Relatively, there were few studies have measured N_2_O and NO emission fluxes simultaneously from Chinese vegetable cropping systems, especially in the greenhouse vegetable cultivations^9^,^23^. It remains unclear whether local conventional input rate of chemical N fertilizer could be reduced to simultaneously attain high yield of vegetable and low N_2_O and NO emissions in greenhouse vegetable cropping systems in China.

We conducted an *in situ* field measurement of annual N_2_O and NO emissions from greenhouse vegetable cropping systems in southeast China. We examined which factors were important to N_2_O and NO emissions in terms of NO/N_2_O ratio or N_2_O plus NO emissions. The main objectives of this study are to quantify seasonal and annual N_2_O and NO emissions in response to chemical N fertilizer application in annual greenhouse vegetable cropping systems. Eventually, this study also attempted to optimize N fertilizer rate for the simultaneous achievements of low N_2_O and NO emissions and high yields in greenhouse vegetable cropping systems in China.

## Results

### N_2_O fluxes

**Figure 1:**
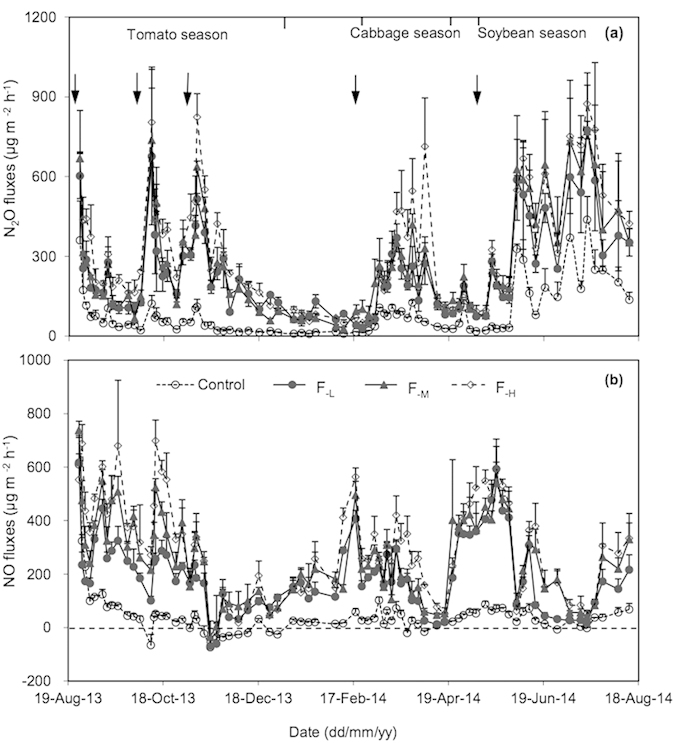
Seasonal dynamics of N_2_O (**a**) and NO (**b**) fluxes from greenhouse vegetable cropping systems over the 2013–2014 annual cycle. Arrows represent basal fertilization and topdressing events. The bars indicate the standard errors of mean (n = 3). F_−L_, F_−M_ and F_−H_ refer to N fertilizer treatments at the low, medium and high application rates, respectively.

Seasonal dynamics of N_2_O fluxes showed similar pattern among the fertilizer treatments (Fig. 1a). In general, N_2_O fluxes followed a sporadic and pulse-like pattern over the whole annual cycle. Substantial N_2_O emissions occurred during the vegetable-growing seasons, while N_2_O fluxes were relatively lower during the inter-cropping fallow periods. The intensive N_2_O flux peaks were mainly observed within one week following basal fertilization and topdressing events accompanied with irrigation (Fig. 1a). Although chemical N fertilizer application did not significantly alter the seasonal pattern of N_2_O fluxes, it greatly increased the magnitude of N_2_O fluxes.

An ANOVA indicated that the N_2_O emissions were significantly affected by cropping type and fertilizer application, while their interactions were not pronounced (Tables 1 and 2). Among the three vegetable cropping seasons, seasonal mean N_2_O fluxes showed the highest for green soybean, while they were relatively comparable between the tomato and Chinese cabbage cropping seasons. For the controls without fertilizer application, N_2_O fluxes averaged 23.90, 39.74 and 115.22 μg N_2_O-N m^−2^ h^−1^ during the tomato, Chinese cabbage and green soybean growing seasons, respectively (Table 1). For the plots with local conventional input level of N fertilizer (F_−M_), seasonal N_2_O fluxes averaged 288.69 μg N_2_O-N m^−2^ h^−1^ during green soybean season, which were 153% and 115% greater than those during tomato and Chinese cabbage seasons, respectively. Over the whole annual cycle, N_2_O emissions totaled 12.45–16.47 kg N_2_O-N ha^−1^ for the fertilizer treatments, of which, about 37–40%, 14–16% and 44–48% were released during the tomato, Chinese cabbage and green soybean seasons, respectively.

**Figure 2:**
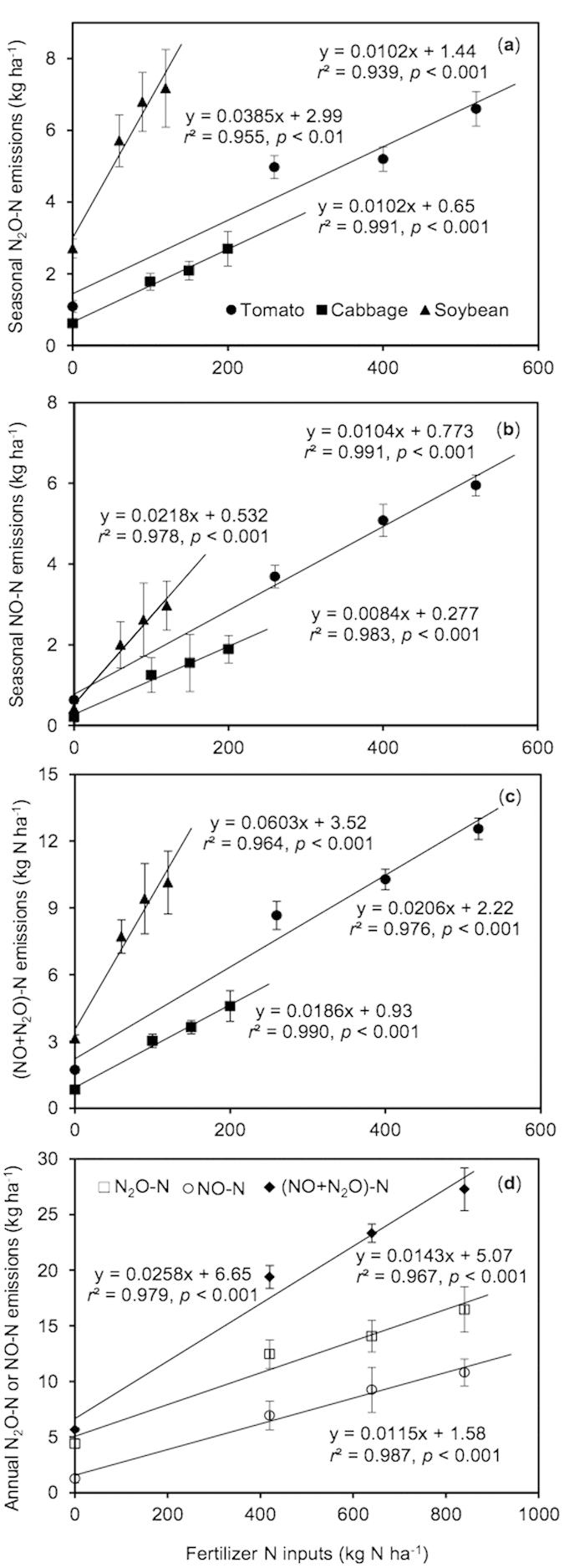
Dependence of seasonal N_2_O (**a**) and NO (**b**) emissions, and annual N_2_O/NO (**c**) emissions on N fertilizer inputs in greenhouse vegetable cropping systems.

Chemical N fertilizer application significantly and consistently increased N_2_O emissions during the three cropping seasons (Tables 1 and 2, Fig. 2). The strongest response of N_2_O fluxes to N fertilizer input was found in green soybean, while the response of N_2_O fluxes to N fertilizer input did not significantly differ between tomato and Chinese cabbage crops (Fig. 2). The parameters in the simulated OLS regressions predicted N fertilizer-induced emission factor of N_2_O to be 1.02% for tomato and Chinese cabbage, and 3.85% for green soybean (Table 1, Fig. 2a). Over the whole annual cycle, the emission factor of N fertilizer for N_2_O was estimated to be 1.43% in the greenhouse tomato-Chinese cabbage-green soybean cropping systems (Fig. 2c). The seasonal background emissions of N_2_O were estimated to be 0.65, 1.44 and 2.99 kg N_2_O-N ha^−1^ for tomato, Chinese cabbage and green soybean, respectively, and thus an annual background emission of N_2_O amounted to as high as 5.07 kg N_2_O-N ha^−1^ (Fig. 2a,c).

### NO fluxes

Similar to N_2_O, seasonal pattern of NO fluxes did not significantly differ among the fertilizer treatments (Fig. 1b). Except the controls without fertilizer application, NO fluxes from the N fertilizer treatments showed a sporadic and pulse-like pattern over the whole annual cycle. Besides substantial NO emissions incurred by N fertilizer application during the vegetable-growing seasons, some NO fluxes were also pronounced during the fallow periods prior to Chinese cabbage and green soybean cropping seasons. Over the whole annual cycle, some peaks of NO flux appeared earlier than those of N_2_O (Fig. 1b).

Seasonal mean NO fluxes did not significantly differ among the three vegetable cropping seasons, although seasonal total NO emissions were significantly affected by cropping type (Tables 1 and 2). For the controls without fertilizer application, NO fluxes averaged 13.82, 13.46 and 17.86 μg NO-N m^−2^ h^−1^ during the tomato, Chinese cabbage and green soybean growing seasons, respectively (Table 1). Seasonal NO fluxes averaged 80.92–85.03 μg NO-N m^−2^ h^−1^, 99.36–111.40 μg NO-N m^−2^ h^−1^, and 121.15–130.48 μg NO-N m^−2^ h^−1^ for the F_−L_, F_−M_ and F_−H_ treatments, respectively. Over the whole annual cycle, NO emissions totaled 1.26 kg NO-N ha^−1^ for the controls, and 6.94–10.81 kg NO-N ha^−1^ for the fertilizer treatments. The tomato, Chinese cabbage and green soybean contributed 50–55%, 17–18% and 27–33% to the annual total NO-N emissions, respectively.

Seasonal total NO emissions were consistently increased with chemical N fertilizer application for the three crops, while the response of NO fluxes to N fertilizer input was stronger in green soybean, relative to tomato and Chinese cabbage cropping seasons (Tables 1 and 2, Fig. 2). On average, the emission factor of NO was estimated to be 1.04% for tomato, 0.84% for Chinese cabbage, and 2.18% for green soybean, respectively (Fig. 2b). Therefore, the annual EF of NO was estimated to be 1.15% in the greenhouse tomato-Chinese cabbage-green soybean cropping systems (Fig. 2c). Over the whole annual cycle, the background NO emission totaled 1.58 kg NO-N ha^−1^ in the greenhouse vegetable cropping systems. Of which, the tomato, Chinese cabbage and green soybean cropping seasons were responsible for 49%, 18% and 33%, respectively (Fig. 2b,d).

### NO-N/N_2_O-N ratio

The ratio of NO-N/N_2_O-N was significantly affected by cropping type and fertilizer application, but it was independent of their interaction (Tables 1 and 2). The ratios of NO-N/N_2_O-N were lowest for green soybean, and highest for tomato among the three cropping seasons. For the controls without fertilizer application, the NO-N/N_2_O-N ratio averaged 0.61, 0.33 and 0.16 during the tomato, Chinese cabbage and green soybean growing seasons, respectively (Table 1). Chemical N fertilizer application consistently increased the NO-N/N_2_O-N ratios cross the three cropping seasons (Fig. 2d). Relative to the controls, chemical N fertilizer application increased the NO-N/N_2_O-N ratios by 21–68%, 118–130% and 145–175% during the tomato, Chinese cabbage, and green soybean seasons, respectively. Over the whole annual cycle, the NO-N/N_2_O-N ratios averaged 0.28 for the controls, and 0.57–0.69 for the N fertilizer treatments.

### Vegetable yield

Vegetable yield (fresh weight) significantly differed among the tomato, Chinese cabbage and green soybean crops, while it was not significantly affected by fertilizer application and the interactions between cropping type and fertilizer input (Tables 1 and 2). Among the fruit-, leaf- and legume-vegetable types, tomato had the highest yield while the yield was the lowest for green soybean. Although chemical N fertilizer slightly increased vegetable yield, this effect was not statistically significant. In particular, no significant difference in vegetable yield among the treatments with different input rates of chemical N fertilizer. Over the whole annual cycle, the highest yield was found for the F_−L_ plots with low input rate of N fertilizer.

### Correlation of N_2_O and NO with soil properties

**Figure 3:**
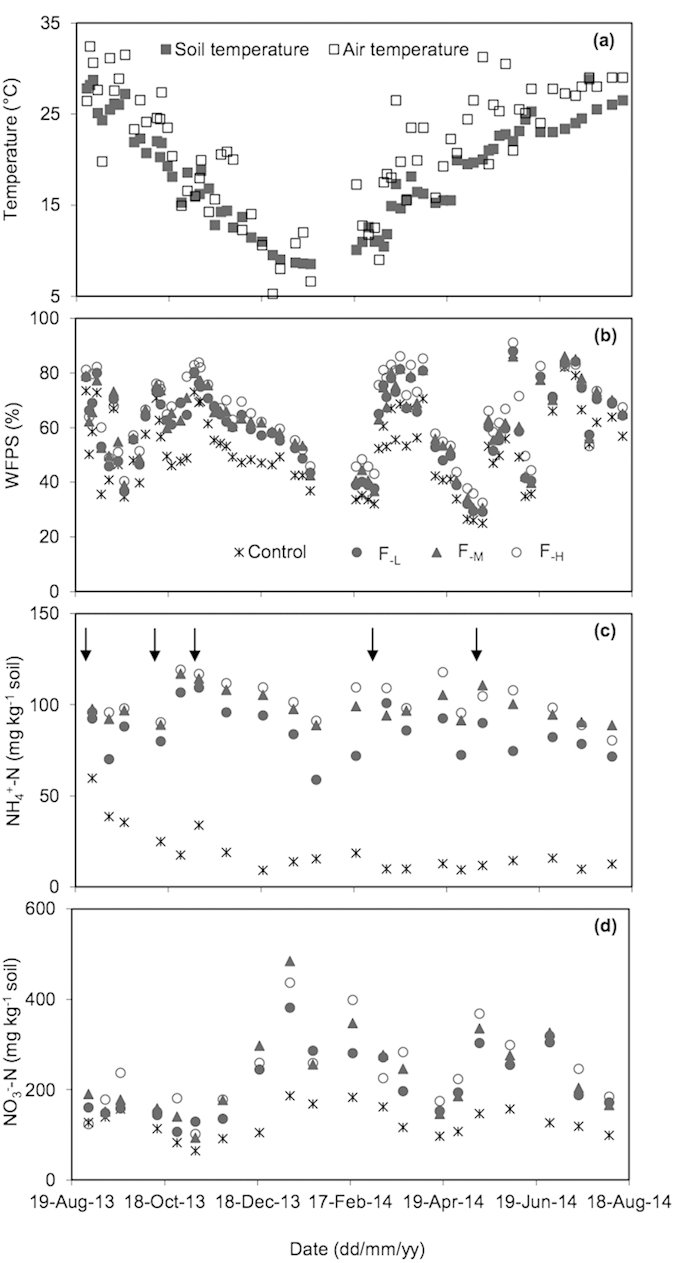
Seasonal dynamics of soil and air temperature (**a**), soil moisture (WFPS, **b**), and soil ammonium (NH_4_^+^-N, **c**) and nitrate (NO_3_^−^-N, **d**) in greenhouse vegetable cropping systems over the 2013–2014 annual cycle. Arrows represent basal fertilization and topdressing events. F_−L_, F_−M_ and F_−H_ refer to N fertilizer treatments at the low, medium and high application rates, respectively.

Over the whole annual cycle, air temperature was slightly higher than soil temperature in the greenhouse except in the winter season through late December to February (Fig. 3a). Both air temperature and soil temperature showed similar seasonal variation pattern, with the highest in summer season and the lowest in winter season. Annual air temperature and soil temperature averaged 21.4 °C and 18.7 °C, respectively. Soil moisture in different treatments showed similar variation pattern over the whole annual cycle, ranging from 24.9% to 91.1% of WFPS (Fig. 3b). In general, soil mineral N contents were increased following fertilization and irrigation events (Fig. 3c,d). For the control plots, soil mineral N contents varied smoothly and were significantly lower than those of the N fertilization plots.

**Figure 4:**
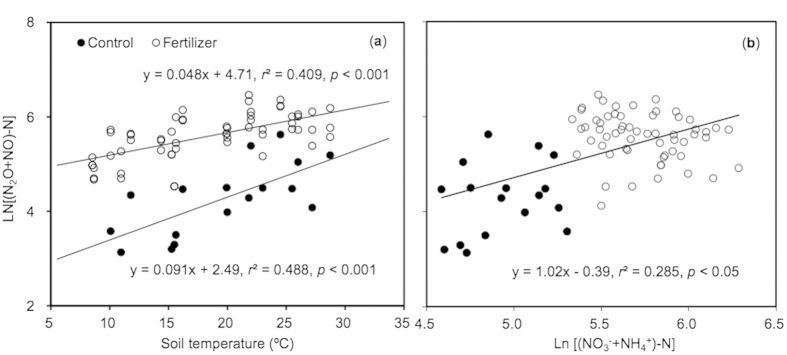
The sum of N_2_O-N and NO-N fluxes dependent on soil WFPS (**a**) and soil mineral N (NH_4_^+^-N+NO_3_^−^-N, b) contents in greenhouse vegetable cropping systems. Both sum of N_2_O-N and NO-N fluxes and soil mineral N contents were log-transformed.

**Figure 5:**
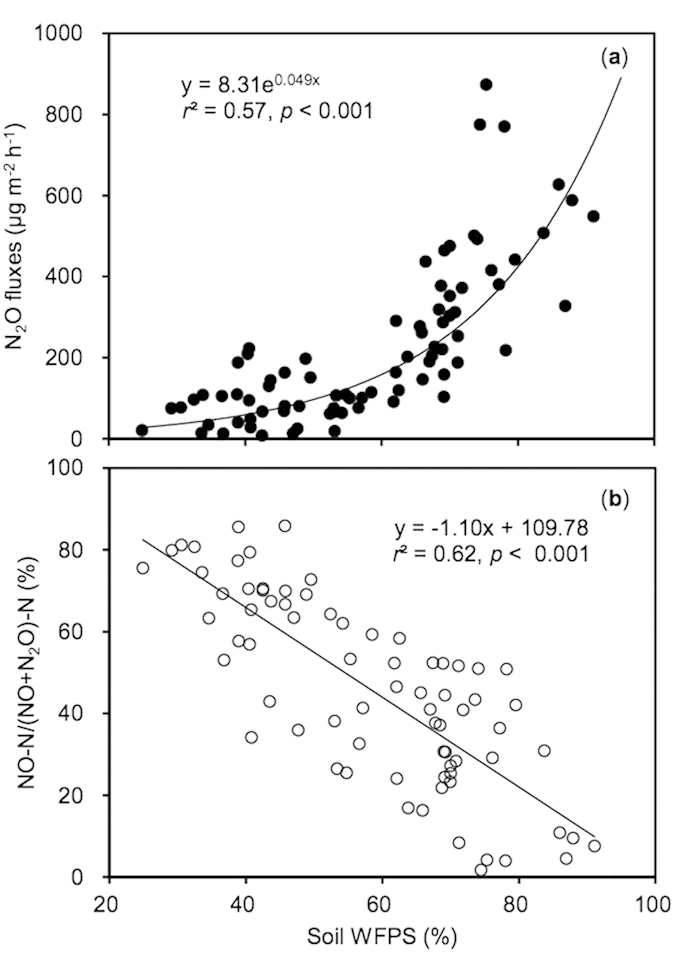
Correlation of soil N_2_O fluxes (**a**) and the ratio of NO-N/(NO+N_2_O)-N (**b**) with soil WFPS in greenhouse vegetable cropping systems.

The N_2_O-N plus NO-N emissions were significantly correlated with soil temperature for the controls and N fertilizer treatments (Fig. 4a). However, the slope of simulated regression was significantly lower for the controls than for the N fertilizer treatments, suggesting chemical N fertilizer application had weakened the response of N_2_O-N plus NO-N emissions to soil temperature (Fig. 4a). The N_2_O-N plus NO-N emissions depended significantly on soil mineral N contents across the treatments (Fig. 4b). Over the whole annual cycle, N_2_O fluxes depended greatly on soil moisture (Fig. 5a). Although NO fluxes were not significantly related to soil moisture over the whole annual cycle, the ratio of NO-N/(NO-N+N_2_O-N) was linearly correlated with soil moisture (Fig. 5b).

## Discussion

To meet the increasing demand of vegetable products in China, greenhouse vegetable cropping systems have been greatly developed in recent decades. Recently, an increasing number studies have focused on N_2_O and NO emissions from Chinese greenhouse vegetables. Consistent with previous studies^9^,^21^,^22^,^23^,^24^, the intensive N_2_O and NO flux peaks generally occurred following fertilizer application accompanied with irrigation during the vegetable-growing seasons. In addition, some peaks of NO flux appeared during the inter-cropping fallow seasons, earlier than the appearance of N_2_O flux peaks, which was primarily due to lower soil moisture suitable for NO emissions.

It is well documented that N_2_O emissions depended significantly on vegetable crop types, which often leads to large inter-seasonal variations in annual greenhouse vegetable cropping systems^9^,^23^,^28^. In the present study, consecutive cultivation of tomato, Chinese cabbage and green soybean constitutes a typical annual fruit-leaf-legume vegetables rotation in greenhouse cropping systems in China. Among the three vegetable cropping seasons, N_2_O emissions showed the highest for green soybean in terms of seasonal mean fluxes or seasonal amount. A similar result was also obtained in an annual greenhouse green soybean-pepper-broccoli vegetables rotation system in China, showing that green soybean contributed the most to the annual total of N_2_O emissions, while NO emissions were comparable between green soybean and broccoli cropping seasons^9^. Indeed, green soybean roots can fix atmospheric N_2_, which can be further transformed into N source for nitrifier and denitrifier to produce N_2_O, and soybean plant itself can also emit large amounts of N_2_O^29^. Higher N_2_O emissions during green soybean seasons relative to tomato and Chinese cabbage seasons might also due to stronger response of N_2_O emissions to N fertilizer application in green soybean than in other vegetable crops (Fig. 2a).

Although N_2_O emissions showed high inter-seasonal variations, annual total of N_2_O emissions in this study was generally comparable to previous results in the greenhouse vegetable cropping systems in China. In the present study, For the N fertilizer treatment, mean annual N_2_O emissions ranged from 12.45 to 16.47 kg N_2_O-N ha^−1^, falling within the range of those (4.20–16.50 kg N_2_O-N ha^−1^) reported in greenhouse vegetable cropping systems in China^9^,^15^,^19^,^21^,^24^,^30^,^31^. The mean fluxes of NO did not show significant inter-seasonal variations, although seasonal total emissions of NO differed among the three cropping types. Over the whole annual cycle, mean NO emissions from N fertilizer treatments ranged from 6.94 to 10.81 kg NO-N ha^−1^, highly close to the most previous estimates in annual vegetable cropping systems in China^32^,^33^. However, our measurements were greater than those estimates obtained by Yao *et al.*^9^, showing annual NO emissions equivalent to be 3.1 kg NO-N ha^−1^ in an annual greenhouse vegetable cropping system in the present study area. The difference in annual NO emissions between the two studies could be associated with divergence in soil pH in these two studies (5.5 *vs*. 8.0). Some studies showed that high soil pH can inhibit NO production during nitrification, and high NO fluxes were frequently observed in soils with low pH^34^,^35^. In addition, a previous measurement taken in the present study area showed that annual NO emissions were as high as 47.1 kg NO-N ha^−1^ in the vegetable fields under farmer’s conventional fertilizer practice^35^, which is remarkably greater than the measurements of this study and some other previous estimates in Chinese vegetable cropping systems.

In the present study, the emission factor of N_2_O was estimated to be 1.43% in annual greenhouse tomato-Chinese cabbage-green soybean cropping systems, comparable to the estimate of 1.10% in an annual green soybean-pepper-broccoli cropping system^9^, but greater than the other recent reports^23^,^24^,^31^,^36^. Based on one year field study in southeast China, the annual N_2_O emission factor was estimated to be 0.38% in the greenhouse tomato-cucumber-celery rotation systems^31^, or 0.36% in the greenhouse red pepper-chrysanthemum vegetable rotation systems^21^. He *et al.*^24^ estimated the emission factor of N_2_O to be 0.27–0.30% in an intensively managed greenhouse tomato cropping system in Northern China. Greater emission factors of N_2_O in this study and Yao *et al.*’s^9^ study than in the other recent vegetable studies was largely attributed to green soybean cropping seasons in greenhouse vegetable systems. Nevertheless, the emission factor of N_2_O in this greenhouse vegetable cropping system was comparable to the earlier estimate of 1.05–1.35% in Chinese upland staple grain crops^37^,^38^,^39^.

Relatively, few studies have estimated emission factor of N fertilizer for NO in croplands. The emission factor of NO averaged 1.15% over the whole annual vegetation rotation cycle in this study, falling within the range of 0.02–3.60% for NO emission factors observed in the vegetable fields worldwide^32^. Li and Wang^33^ estimated the emission factor of NO to be 2.4% in an annual Chinese cabbage cropping field in the Pearl River Delta, China, greater than the estimates of this study. However, our estimated values were significantly greater than the reported value of 0.05% in annual rice-wheat cropping rotation systems^40^, or 0.36% in annual greenhouse green soybean-pepper-broccoli cropping systems in the present study region. Yan *et al.*^39^ estimated the average emission factor of NO to be 0.71% for global upland grain croplands, which is slightly lower than our estimates in greenhouse vegetable cropping systems. By taking N_2_O and NO emissions into account together, the emission factor of N fertilizer was estimated to be 2.58% in annual greenhouse vegetable cropping systems (Fig. 3c). Nevertheless, more field measurements are highly needed in typical cropping systems given that the emission factors of N_2_O and NO are documented to be associated with environmental factors, soil properties and agricultural practices^9^,^22^,^31^,^32^.

A great many studies have documented high background emission of N_2_O in greenhouse vegetable cropping systems in China. In the simulated linear regression of N_2_O emissions with N fertilizer application rates (Fig. 2a,c), the background emission of N_2_O was, on average, estimated to be 5.07 kg N_2_O-N ha^−1^ over the whole annual cycle. The estimate of annual N_2_O background emissions in this study was highly close to some previous measurements taken in the present study area. Annual background emissions of N_2_O were estimated to be 3.40, 5.0, and 5.65 kg N_2_O-N ha^−1^ in the greenhouse vegetable fields with annual cropping rotations of tomato-cucumber-celery, green soybean-pepper-broccoli and red pepper-chrysanthemum, respectively^9^,^21^,^31^. In contrast, annual background emissions of NO averaged 1.58 kg NO-N ha^−1^ in this study, greater than previous estimates of 0.16–0.40 kg NO-N ha^−1^ by Mei *et al.*^32^, 0.41 kg NO-N ha^−1^ by Deng *et al.*^35^ or 0.41 kg NO-N ha^−1^ by Yao *et al.*^9^ in vegetable cropping systems in this region.

Although relatively few studies have focused on annual background emissions of N_2_O and NO in the typical greenhouse vegetable fields, several available studies suggested that background emissions of N_2_O and NO in greenhouse vegetable fields were generally greater than those in grain staple croplands in China^9^,^21^,^24^,^31^. Background N_2_O emissions were estimated, on average, to be 1.22–1.87 kg N_2_O-N ha^−1^ yr^−1^ in Chinese grain croplands^39^,^41^,^42^,^43^. Greater background N_2_O and NO emissions from greenhouse vegetable fields relative to grain staple croplands might be associated with frequent irrigation coupled with residual N from heavy fertilizer inputs for several years, and/or run-off from adjacent heavily fertilized plots. Nevertheless, available studies suggest that background N_2_O and NO emissions from greenhouse vegetable cropping systems could play particularly important roles in developing a national inventory of N_2_O and NO emissions from vegetable fields in China.

In general, N_2_O and NO are primarily produced during soil nitrification and denitrification processes^2^,^44^, which are highly associated with soil properties in agricultural fields^2^,^23^,^27^,^45^. Consistent with previous studies^9^, the N_2_O-N plus NO-N emissions depended on soil mineral nitrogen availability and temperature in this study (Fig. 4). Moreover, the response of N_2_O-N plus NO-N emissions to soil temperature was stronger in the control than in N fertilizer application treatments. A similar result showed that fertilizer application increased the response of N_2_O-N plus NO-N emissions to soil mineral N availability, but decreased their response to temperature in greenhouse vegetable cropping systems^9^. This suggests that N_2_O-N plus NO-N emissions were primarily limited by soil mineral N availability.

Soil moisture was lower in the control than fertilizer treatments during the tomato and Chinese cabbage cropping seasons, but there were no obvious differences in soil moisture among all treatments during the green soybean season (Fig. 3b). Relative to green soybean, tomato and Chinese cabbage require more water resource, and thereby tomato and Chinese cabbage crops would uptake more water from soils under fertilizer application. In contrast to previous studies^9^, the dependence of N_2_O-N plus NO-N emissions on soil WFPS was not significant in this study. Instead, N_2_O fluxes were positively related to soil WFPS, while the ratio of NO/(NO + N_2_O) was negatively correlated to soil WFPS over the whole annual vegetable cropping cycles (Fig. 5). The emission ratio of NO/N_2_O or NO/(NO+N_2_O) has been frequently used as an indicator of the relative importance of nitrification and denitrification in producing NO and N_2_O^46^,^47^,^48^. The simulated regression projected that NO would exceed N_2_O emissions when soil WFPS was lower than 54% in greenhouse vegetable cropping systems, highly in agreement with the results obtained in aggrading forests^49^.

Excessive chemical N fertilizer application in vegetable fields in China has been frequently pointed out, which would incur substantial N_2_O and NO emissions. Among the N fertilizer treatments, the F_−L_ treatments with low N fertilizer input rate showed the highest yield and lowest N_2_O and NO emissions, suggesting that local conventional input rate of chemical N fertilizer could be reduced by one-third to attain high yield of vegetable and reduce N_2_O and NO emissions in greenhouse vegetable cropping systems in China. With the rapid development of greenhouse vegetable cropping systems in China, nevertheless, it is urgent to establish fertilizer management optimization strategies specific to greenhouse vegetables production so as to simultaneously improve vegetable production and mitigate greenhouse gases emission in China.

## Methods

### Experiment site

Field experiments were conducted in Nanjing vegetable production farm located at suburban Nanjing, Jiangsu province, China (32°04′N, 118°58′E). The experimental region is characterized by a monsoon climate with annual mean temperature of 17.8 °C and precipitation of 1090 mm over the annual experimental cycle. The experimental site is dominated by conventional open-air and plastic greenhouse vegetable cropping fields. Soils at the experimental field are classified as silt loam, consisting of 15.2% sand, 30.4% silt and 54.5% clay with an initial pH of 5.5 (1:2.5, water/soil, w/w) and an average bulk density of 1.13 g cm^−3^. Total N and organic C contents were 1.9 g kg^−1^ and 14.7 g kg^−1^, respectively.

### Field experiments

In the 2013–2014 annual cycle, field experiment plots were established in the greenhouse vegetable cropping systems. Three parallel greenhouse vegetable cropping field blocks with an over 10-year history of continuous vegetable cultivation were selected as experimental replicates, which were identically covered with polyethylene plastic film and had no extra lighting or heating. Each replicated greenhouse comprised of four experimental treatments, referring to the controls without fertilizer application (Control), and the treatments with chemical N fertilizer applied at low (F_−L_, about two-third of the farmer’s conventional nitrogen input), medium (F_−M_, farmer’s conventional nitrogen input), and high (F_−H_, four-third of the conventional nitrogen input) rates. The two-paired gas-sampling plots for each treatment were randomly established in each 5 × 80 m^2^ grid of greenhouse vegetable field. In total, each treatment had 6 gas-sampling plots, and each field plot was 1.8 × 1.8 m^2^. The individual plots were separated by protection rows that were 0.5 m in width.

In line with local cropping rotations for greenhouse vegetable production systems, three vegetable crops, namely, tomato (*Solanum lycopersicum*), Chinese cabbage (*Brassica chinensis*), and green soybean (*Glycine max*) were consecutively cultivated, which constituted an annual fruit-leaf-legume-vegetables cropping rotation pattern. For the fertilizer treatments, compound fertilizer (N: P_2_O_5_: K_2_O = 15%: 15%: 15%) was used as the N fertilizer. According to the local farmer’s practice, basal N fertilizer was broadcasted on the soil surface and then incorporated into the soil by plowing. At the topdressing events, N fertilizer was first dissolved in the water and then flushed to the field with irrigation for the fertilizer plots, and the control plots were irrigated only with similar amount of water. Other management practices, including the fertilization time, irrigation and tillage, were conducted according to the local practices of the farmer. The information about the cultivation and fertilization events was detailed in Table 3.

### N_2_O and NO fluxes measurement

Fluxes of N_2_O and NO were simultaneously measured using the static opaque chamber-gas chromatograph (GC) method as described in Liu *et al.*^21^ and Yao *et al.*^9^. Flux measurements were taken over the period of 26 Aug 2013 to 13 Aug 2014 (353 days) in the greenhouse vegetable cropping systems. A PVC flux collar (50 × 50 × 15 cm) was pre-installed in the middle of each plot before vegetables transplanting or sowing. The top edge of the collar had a groove (5 cm in depth) filled with water to seal the rim of a chamber during gas collection. The sampling chambers were made of opaque PVC materials at a size of 50 cm in height (or 100 cm in height depending on vegetable growth) ×50 cm in width ×50 cm in length. The chamber was wrapped with a layer of sponge and aluminum foil to minimize air temperature changes inside the chamber and equipped with a circulating fan to ensure complete gas mixing during the period of sampling. There was no difference in the vegetable planting density between inside and outside the chamber. Over the whole annual cycle, gas samples were taken twice a week, except that they were taken once every one or two days for one week following fertilizer application. Gas samples were collected between 0800 and 1000 local standard time on each sampling day. A 1.5-L gas sampling bag (Delin Gas Packing Co., LTD, Dalian, China) was used to take gas samples from the headspace at 0, 5, 10, 15 and 20 min after the chambers closure, and stored for laboratory analysis within a few hours. The chamber headspace temperature was recorded for gas density correction in flux calculation using a thermometer.

The mixing ratios of N_2_O were quantified by a gas chromatograph (Agilent 7890A, USA) equipped with an electron capture detector (ECD), which was detailed in our previous studies^21^,^50^,^51^. A gas mixture of argon-methane (5%:95%) as N_2_O carrier gas was at a flow rate of 40 mL min^−1^. The column and ECD detector temperatures were maintained at 40 °C and 300 °C, respectively. The mixing ratio of NO was analyzed with a model 42*i* chemiluminescence NO-NO_2_-NOx analyzer (Thermo Environment InstrumentsInc., USA), which was calibrated once every two or three months using the calibration system from the same manufacture and the standard gas from the National Center of Standard Matters (Beijing, China). A nonlinear fitting approach was adopted to determine N_2_O and NO fluxes, as described by Kroon *et al.*^52^. Mean of fluxes taken from the paired plots represent flux measurements of the treatment within each greenhouse. Seasonal and annual cumulative N_2_O and NO emissions were sequentially accumulated from the emissions between every two adjacent intervals of the measurements.

### Auxiliary measurements

Soil temperature and moisture (0–10 cm) were monitored when gas samples were collected using a portable rod probe (MPM-160). Soil moisture was further converted into water filled pore space (WFPS) by the following equation: WFPS = (soil volumetric water content/(1 – (soil bulk density/2.65)) ×100%). Here, 2.65 Mg m^−3^ was the assumed soil particle density^21^. Soil samples were collected prior to experiment establishment to determine background information of topsoil (0–15cm) physiochemical properties. Soil bulk density was measured using a 100 cm^3^ cylinder that was pressed into the soil. Soil pH was determined in a volume ratio of 1:2.5 (soil/water) with a compound glass electrode (PHS-3 C mv/pH detector, Shanghai, China). Over the whole annual cycle, soil samples at 0–15 cm depth were collected every 10–15 days for soil mineral N (NO_3_^−^ and NH_4_^+^) analysis. According to the Chinese Soil Society Guidelines^53^, soil NO_3_^−^-N contents were measured following the two wavelength ultraviolet spectrometry at 220nm and 275nm, and NH_4_^+^-N contents were measured using indophenol blue method (HITACHI, U-2900, Japan).

### Statistical Analysis

Differences in seasonal cumulative N_2_O and NO emissions, NO-N/N_2_O-N ratio and vegetable yield as affected by cropping type, N fertilizer and their interactions were examined using a two-way analysis of variance (ANOVA). Linear or nonlinear regression analyses were conducted to examine the dependence of N_2_O and NO emissions on soil physiochemical parameters. A linear regression model with the character of Ordinary Least Squares (OLS) was used to fit N_2_O-N and NO-N emissions by nitrogen inputs (N) on seasonal and annual scales, in which the simulated slop and constant represented the emission factor of chemical N fertilizer for N_2_O (EF) and background emission of N_2_O, respectively. All statistical analyses were performed using JMP software version 9.0.2 for Windows (SAS Inst., NC, USA, 2010).

## Additional Information

**How to cite this article**: Zhang, Y. *et al.* Response of nitric and nitrous oxide fluxes to N fertilizer application in greenhouse vegetable cropping systems in southeast China. *Sci. Rep.* **6**, 20700; doi: 10.1038/srep20700 (2016).

## Figures and Tables

**Table 1:** Seasonal and annual total (Mean ± 1SE, n = 3) of N_2_O and NO emissions and vegetable yield (fresh weight) in greenhouse vegetable cropping systems.

**Measurement period**	**Treatment**	**N fertilizer**	**N**_**2**_**O-N**	**NO-N**	**NO-N/N**_**2**_**O-N ratio**	**Yield (t ha**^**−1**^)
		**(kg N ha**^**−1**^)				
Tomato (24 Aug 2013~3 Mar 2014, 190 days)	Control	0	1.09 ± 0.17d	0.63 ± 0.09fgh	0.61 ± 0.14abcd	112.40 ± 5.38a
	F_−L_	260	4.97 ± 0.32abc	3.69 ± 0.28b	0.74 ± 0.04abc	120.67 ± 5.46a
	F_−M_	400	5.20 ± 0.34abc	5.08 ± 0.40a	0.98 ± 0.17a	123.07 ± 5.73a
	F_−H_	520	6.60 ± 0.48a	5.95 ± 0.26a	0.91 ± 0.08ab	122.40 ± 9.83a
Chinese cabbage (4 Mar~7 May 2014, 65 days)	Control	0	0.62 ± 0.02d	0.21 ± 0.12h	0.33 ± 0.03cd	71.41 ± 8.57b
	F_−L_	100	1.78 ± 0.23cd	1.25 ± 0.43efgh	0.72 ± 0.11abc	79.84 ± 6.50b
	F_−M_	150	2.09 ± 0.26cd	1.55 ± 0.71defg	0.76 ± 0.16abc	73.17 ± 4.52b
	F_−H_	200	2.70 ± 0.48bcd	1.89 ± 0.34cdef	0.73 ± 0.07abc	69.16 ± 4.72b
Green soybean (8 May~13 Aug 2014, 98 days)	Control	0	2.71 ± 0.26bcd	0.42 ± 0.16gh	0.16 ± 0.02d	8.39 ± 1.07c
	F_−L_	60	5.71 ± 0.72ab	2.00 ± 0.57cde	0.38 ± 0.10cd	10.25 ± 1.36c
	F_−M_	90	6.79 ± 0.82a	2.62 ± 0.91bcd	0.43 ± 0.09bcd	9.26 ± 0.92c
	F_−H_	120	7.17 ± 1.08a	2.97 ± 0.61bc	0.43 ± 0.05bcd	9.94 ± 0.57c
Annual cycle (24 Aug 2013~13 Aug 2014, 353 days)	Control	0	4.41 ± 0.45	1.26 ± 0.37	0.28 ± 0.03	192.20 ± 15.01
	F_−L_	420	12.45 ± 1.27	6.94 ± 1.28	0.57 ± 0.09	210.76 ± 13.31
	F_−M_	640	14.07 ± 1.42	9.25 ± 2.02	0.69 ± 0.14	205.50 ± 11.17
	F_−H_	840	16.47 ± 2.04	10.81 ± 1.21	0.66 ± 0.04	201.51 ± 15.13

Data shown are means ± standard errors of three replicates. The flux measurements period includes the vegetable-growing season and the following fallow period. Different letters within the same column indicate significant differences among treatments at *p *< 0.05 level.

**Table 2:** A two-way ANOVA for seasonal N_2_O and NO emissions, NO-N/N_2_O-N ratio and yield as affected by cropping type and N fertilizer application in greenhouse vegetable cropping systems.

**Factors**	***df***	**N**_**2**_**O-N**			**NO-N**			**NO-N/N**_**2**_**O-N ratio**			**Yield**		
		***SS***	***F*****-ratio**	***P*****-value**	***SS***	***F*****-ratio**	***P*****-value**	***SS***	***F*****-ratio**	***P*****-value**	***SS***	***F*****-ratio**	***P*****-value**
Cropping type (C)	2	91.30	32.8	<0.001	43.14	114.9	<0.001	1.33	22.2	<0.001	73458	418.4	<0.001
Fertilizer (F)	3	81.91	19.6	<0.001	52.51	93.2	<0.001	0.71	7.9	<0.001	184.08	0.7	0.56
C × F	6	11.98	1.4	0.24	12.53	11.1	<0.001	0.08	0.4	0.84	230.41	0.4	0.85
Model	11	185.18	12.1	<0.001	108.18	52.4	<0.001	2.12	6.4	<0.001	73872	76.5	<0.001
Error	24	33.42			4.51			0.72			2107		

**Table 3:** Vegetable cultivation and N fertilization practices in annual greenhouse vegetable cropping systems.

**Cropping system**			**N Fertilization (kg N ha**^**−1**^)				
**Cropping type**	**Transplanting/Sowing**	**Harvest**	**Date**	**Type**	**F**_**−L**_	**F**_**−M**_	**F**_**−H**_
Tomato	24 Aug 2013	09 Jan 2014	23 Aug 2013	Basal	130	200	260
			10 Oct 2013	Top dressing	78	120	156
			04 Nov 2013	Top dressing	52	80	104
Chinese cabbage	04 Mar 2014	20 Apr 2014	28 Feb 2014	Basal	100	150	200
Green soybean	10 May 2014	22 Jul 2014	05 May 2014	Basal	60	90	120

F_−L_, F_−M_ and F_−H_ represent N fertilizer treatments at the low, conventional and high application rates, respectively.
